# Substances use and its association with socio-demographic, family, and environment-related factors among technical and vocational education and training college students in Ataye, Ethiopia; an institution-based cross-sectional study

**DOI:** 10.1186/s12889-020-09797-w

**Published:** 2020-11-11

**Authors:** Abate Dargie Wubetu, Sintayehu Getachew, Wassie Negash

**Affiliations:** 1 Department of Nursing, College of Health Science and Medicine, Debre Berhan University, P.O. Box 445, Debre Berhan, Ethiopia; 2 Ataye General Hospital, North Shewa Zone, Addis Ababa, Amhara Ethiopia

**Keywords:** Prevalence, Lifetime, Last month, Substance use, College students

## Abstract

**Background:**

Legal substances use is prevalent in Ethiopia. Substance use can have several health problems that are potentially harmful to educational performance, social issues, psychological and physical wellbeing. This study aimed to know the prevalence of lifetime and last month’s substance use and its associated factors among technical and vocational education and training College students in Ataye town.

**Methods:**

An institution-based cross-sectional study was conducted from 1 March to last May 2019. Participants were selected using a simple random sampling technique. Data collected by using a structured and pretested interviewer-administered questionnaire. Data collected by five trained diploma nurses with close supervision. Odds ratio with their 95% confidence interval, and *p*-value less than 0.05 used to declare the statistical significance of associated factors.

**Results:**

Four hundred eighty-three individuals participated in the study, which was a response rate of 94%. The prevalence of lifetime legal substance use was 32.5% (95% CI: 28.2, 36.5). The prevalence of last month’s legal substances use was 21.9% (95% CI = 18.2, 25.5). Among lifetime legal substance users, the majority (25.5%) chewed khat. The others, (19.5%) drunk alcohol, and, 15.3% smoked cigarettes in a lifetime. Lifetime cannabis and cocaine users were 2.5, and 7.2% respectively. Among last month’s legal substance users, (21.9%) chewed khat followed by alcohol drinking (16.6%), and cigarette smoking (15.3%). In the last month, 1.2 and 3.3% of students used cannabis and cocaine respectively. Multivariate logistic regression showed that being male, having a divorced family, living greater than 20 years in the town, having substance user family members, having intimate friend substance users, and easy availability of substances were independent predictors of lifetime legal substances use.

**Conclusions:**

The prevalence of last month and lifetime legal substance use at Ataye Technical and Vocational Education and Training **(**TVET**)** college students were analogous with most studies done in Ethiopia. It is advisable if the college administrators work together with town administrators to mitigate the problem including closing substance use houses around the school. Overall, Substance use among adolescents should get further emphasis to lower the prevalence.

## Background

Substance use has the capability of affecting the state of the body and the mind by either depressing or stimulating the central nervous system or producing other biochemical harmful effects [1–3]. An estimated 5 % of the global adult population use drugs at least once in their lifetime as studied in 2015. The more worrisome fact is about 0.6% of the global adult population suffers from drug use disorders [4].

World drug report revealed that more than a quarter of a billion people use drugs globally. Roughly, almost half a million people died because of drug use in 2015, according to the World Health Organization (WHO). Of those deaths, nearly 170,000 (2.7%) deaths directly associated with drug use disorders (mainly overdoses), [5].

According to the Ethiopian Demographic and Health Survey (EDHS) report, one-third of women and about ha1f of men reported drinking alcohol at some point in their lives. The proportion of legal substances use is increasing every year in both sexes. The use of tobacco increases with the age of men. There is a wide regional variation in cigarette smoking in Ethiopia [6].

Drug use is associated with adverse health consequences. Problems of substance use seem to be a rapidly growing concern globally. It is also a major threat among youth in college and university. Some of the researchers have shown that the use of drugs by school-going youth does not only decrease their academic performance; but also makes them vulnerable to crime. Furthermore, drug use exposes them to health risks among other numerous problems [7–13].

Substances use is a common phenomenon among students in Ethiopia. It has also been noted that family background, student pocket money, peer pressure, accessibility of substances, and customs of society contribute to the increased rate of substance use among college students. Most students, staffs at an institution of higher education, and youths in the community considered are at high risk of substance use, [14–20].

The problem of substance use has historically been linked to Ataye town due to the accessibility of substances. Khat use is a common phenomenon in the study area [17]. Even though such problems are one of the top health risks among college students, there are no adequate studies conducted to explore the prevalence and associated factor of a lifetime and last month’s substance use in technical and vocational education and training (TVET) college students. The findings of this study will be useful to the education bureau and school administrations to develop strategies to mitigate students’ substance use behavior. The study findings also may help curriculum developers in formulating and incorporating psycho-education programs in TVET College that address the risk of substance use. School managers also may benefit from findings so that they may come up with policies and strategies for controlling this potentially dangerous habit.

The main aim of this study was to assess the lifetime and last month prevalence of substance use and to identify associated factors with it. Each type of substance use proportions was also described.

## Methods

### Study area

The study was conducted at Ataye TVET College. Ataye TVET College was found in the North Showa zone that found in Amara Regional state of Ethiopia. The study area is located 272 km from Addis Ababa and 130 km from Debre Berhan town. Orthodox, Muslim, and protestant followers are the major inhabitants found in the study area. At Ataye TVET College, there are 1433 students enrolled in the academic year. Out of these, 683 were male, and 750 were female students.

### Study design, and period

An institution-based cross-sectional study was conducted from 1 March to last May 2019.

### The study participants

There are nine departments from the first year to the third year. The departments are Agriculture, hotel kitchen operation, Electricity, Auto engine service, Garment, Surveying, Construction, Metalwork, and Road construction. All regular Ataye TVET college students, and who were available during the study period considered as the study population. Students could not reach in three visits during the data collection period excluded from the study.

### Study variables

#### Dependent variables

Lifetime and last month substance use.

#### Independent variables

Socio-demographic characteristics: (age, sex, residence, economic status, education status, religion, ethnicity, and, living status, mother education level, father education level, and having substance user friend, lack of family supervision, family conflict, family history of substance use, family income, availability of substance, source of money, grade level, peer pressure, marital status of the family.

### Sample size determination

The sample size was calculated using a single population proportion formula. A 15.36% rate of substance use was taken from the related study [17], with a margin of error 5%, confidence level 95%, and non-response rate 10%. The final sample size became 514 students.

### Sampling technique, and procedure

Out of nine departments, five departments (55%) selected by using the lottery method. Ataye TVET College has 1433 students enrolled in the study academic year. The study participants selected by using a simple random sampling technique by generating random numbers using Open EPI software.

### Method of data collection, and tools

Data were collected by using a structured and interviewer-administered questionnaire. The questionnaire included variables like a lifetime and last month’s substance use, socio-demographic, and economic factors, the practice of substance use, the reason for substance use, and family history of substance use. The questionnaire of independent factors was assessed by using questionnaires adapted from reviewing similar related articles. The outcome variables (lifetime, and last month substance use) were assessed by using the first two sections of the English version of Alcohol, Smoking, and Subtance Involment Screening Test (ASSIST) tool [21] “yes”/ No questions. Lifetime substance use assessed by asking “Have you ever use one of the following substances, and if yes which type of substance use you can circle more than one option?”. Last month substance use was also assessed by asking, “have you use one of the following substances in the last 30 days, and if yes, which type of substance; you can circle more than one option?”

### Data quality assurance

The questions were translated into the local language (Amharic). To keep the quality of data, data collectors and supervisors trained for 1 day regarding the necessary explanation about the current research. Data collected by five trained diploma nurses with close supervision. A pre-test was conducted on 5% (*n* = 24) students among similar study populations from non-participating departments before 2 weeks of the actual data collection period. After the pretest, the wording and the sharpness of the questions were modified. The collected data were reviewed and checked for completeness before data entry. The data properly coded and entered into Epi Info 3.5.1 and exported to SPSS V. 21 for analysis.

### Operational definitions

#### Lifetime legal substance use

Use of one or more legal substances (Ethiopia context) for nonmedical purposes (alcohol, khat, and cigarette) after joining the Ataye TVET College.

#### Last month legal substance use

Use of one or more legal substances (Ethiopia context) for nonmedical purposes (alcohol, khat, and cigarette) in the past 30 days before the data collection period.

#### Legal substances

Drugs are not prohibited from selling, buying, and using among above 18 years old persons (alcohol, khat, and cigarette) during the study period.

#### Illegal drugs

Drugs, which prohibited from selling, buying, and using among all age groups (cocaine and cannabis) during the study period.

### Data processing, and analysis

Lifetime and last month’s legal substance use were the dependent variables. Each substance use in a lifetime and last month were investigated and discussed in the discussion section. Illegal substance use prevalence was assessed as additional information. Socio-demographic, and economic factors, the practice of substance use, the reason for substance use, and family history of substance use were the independent variables of the study. The missed data were not encountered in this study. Variance Inflation Factor (VIF) was calculated for each collinear variable and its value ranges from two up to three. Bivariate comparison and statistical significance of differences were tested by the chi-square test. Bivariate and multivariate binary logistic regression analysis was performed to identify associated factors. The backward logistic regression method was used. Odds ratio with their 95% confidence interval and *p*-value less than 0.05 considered to declare the statistical significance of associated factors.

### Ethical consideration

Ethical clearance obtained from the Debre Berhan University ethical review committee. A letter of cooperation wrote to each study institution, and a permission letter taken from the study institutions. The data collectors took oral informed consent from each study participant, whose age is 18 years and above. Moreover, the data collectors took assent from participants whose age was less than 18 years. Written informed consent taken from participants’ family/legal guardian whose age was less than 18 years.

## Results

### Prevalence’s of substances use

Four hundred eighty-three individuals participated in the study, which was a response rate of 94%. The prevalence of lifetime legal substance use was 32.5% (95% CI: 28.2, 36.5). The prevalence of last month’s legal substances use was 21.9% (95% CI = 18.2, 25.5). Among lifetime legal substance users, the majority (25.5%) chewed khat. The others, (19.5%) drunk alcohol, and, 15.3% smoked cigarettes in a lifetime. Lifetime cannabis and cocaine users were 2.5, and 7.2% respectively. Among last month’s legal substance users, (21.9%) chewed khat followed by alcohol drinking (16.6%), and cigarette smoking (15.3%). In the last month, 1.2 and 3.3% of students used cannabis and cocaine respectively.

### Sociodemographic characteristics of students

Four hundred eighty-three students participated in the study, which was a response rate of 94%. The median age of the students was 20 years with an interquartile range of three, (Q1 = 18, Q3 = 21 years). Among the socio-demographic variables, a nearly equal proportion was observed in terms of sex (49.7% male and 50.3% female). The majority, (71.6%) students were orthodox in religion and the remaining (28.4%), and (4.1%) were Muslim and protestant respectively. The higher proportion, (60.5%) of students were from the Amhara region and the remaining were from Tigray, (18%) and, (12%) from the Oromia region. Almost half of the students, (47%) were level-I in academics. The remaining (14.7%), (26.3%), and (12%) of students were Level, II, III, IV in academic level respectively. Almost, a similar proportion of students lived alone (42.4%), and with family (40.2%). Others, (17.4%) lived with peers. Almost 69 % of students, (68.5%) earn average monthly pocket money of greater than 200 ETB (Ethiopian Birr) and the left earn less than 200 ETB. The highest proportion of students, (95.4%) were from an urban area and, (4.6%) from a rural area.

### Family-related factors of lifetime legal substances used

Almost half, (57.4%), and (52%) of student’s mother and father not attended modern education respectively. Sixty percent of student’s parents lived in an urban area, and (44%) was a farmer in occupation. Nearly 80 % of students’ parents were married and nearly 40 %, (43.1%) of student’s parents lived for 1–5 years in the Ataye town (Table 1).

**Table 1:** Family-related factors of lifetime legal substances use among Ataye TVET students, north Shoa zone, Ethiopia, 2019

Variables	Lifetime substance use		***P***-value
	Yes (%)	No (%)	
Mother’s educational level			
No education	75 (15.6)	202 (41.8)	0.001
Grade 1–12	42 (8.6)	71 (14.8)	
College	40 (8.2)	53 (11)	
Father’s educational level			
No education	65 (13.5)	186 (38.5)	0.003
Grade 1–12	39 (8.1)	68 (14)	
college	53 (11)	72 (14.9)	
Family residency			
Rural	70 (14.5)	123 (25.5)	0.150
Urban	87 (18)	203 (42)	
parents job			
Government	47 (9.7)	122 (25.3)	0.004
Farmer	63 (13)	149 (31)	
Merchant	47 (9.7)	55 (11.4)	
Family monthly income (ETB)			
< 1000	21 (4.4)	87 (18)	0.001
1001–1500	26 (5.4)	90 (18.6)	
1501–2000	17 (3.5)	60 (12.5)	
> 2000	93 (19.3)	89 (18.4)	
Parent marital status			
Married	88 (18.2)	208 (43.1)	0.001
Widowed/widower	11 (2.3)	19 (3.9)	
Divorced	58 (12)	19 (3.9)	
Years living in the town			
1–5	70 (14.5)	138 (28.6)	0.001
6–10	19 (3.9)	77 (16)	
11–15	7 (1.5)	33 (6.8)	
16–20	29 (6)	63 (13)	
> 20	32 (6.6)	15 (3.1)	
Having substance user family			
Yes	72 (14.9)	20 (4.1)	0.001
No	85 (17.6)	306 (63.4)	

### Environmental factors of substances use

**Fig. 1:**
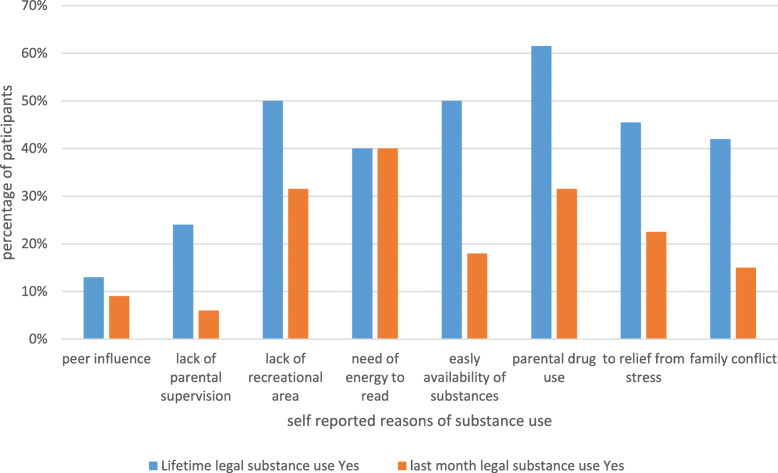
Self-reported environmental reasons for lifetime legal substance use among Ataye TVET students, Ethiopia, in 2019

There are many houses opened by merchants in the study area for substance use in-group. The majority, (90%) of TVET students reported, “They used both the legal and illegal substances at substance use houses.” The remaining reported they use the substances with their family at their own living houses. Among the total students, (61.5%) reported that they start to use legal substances in their lifetime due to easily available in their hometown. Similar proportions (40%) of both lifetime and last month legal substance users reported “They started substance use due to need of energy to read” academic issues. Students reported additional environmental factors for their lifetime and last month’s legal substance use, (Fig. 1).

### Lifetime and last month legal substance use and its correlates

The lifetime prevalence of legal substance use was 32.5% (95% CI: 28.2, 36.5). Among lifetime users, the majority (25.5%) chewed Khat; followed by alcohol drinking (19.5%). Students in different age groups did not equally experience legal substance use in a lifetime. Being from the urban and rural areas had not contributed to both lifetime and last month’s legal substance use. However, other predictor variables were statistically significant with the association of lifetime alcohol, khat, and cigarette use. This implied that students in different categories of the variables were not equally practiced the use of the listed legal substances in a lifetime.

The prevalence of last month’s substance use was 21.9% (95% CI = 18.2, 25.5). Among last month’s substance users, the same proportions (21.9%) chewed khat; followed by alcohol drinking, (16.6%), and cigarette smoking, (15.3%).

Alcohol, khat, and cigarette use in the last month and its variation across study subjects’ characteristics compared using chi-square with its *p*-value. The result of this study showed that being in different age groups had a contribution to the prevalence of last month’s licit substance use. Being from urban and rural in residency was statistically insignificant for all the three listed drugs (alcohol, khat, and cigarette). This means students in these subcategories equally practiced substances use in the last month. Living alone and living with others had a contribution to the variation of last month’s prevalence of alcohol drinking (*p*-value = 0.02) and Khat chewing (*p*-value = 0.001), but not for cigarette smoking (*p*-value = 0.2). All the reported predictor variables of the students were statistically significant correlates with last month’s substance use. This indicated that students with different predictor variables were not equally practiced drugs in the last month (Table 2).

**Table 2:** Lifetime legal drugs use and its correlation with students and family characteristics among Ataye TVET college students, Ethiopia, 2019

Variables	Lifetime alcohol use		Lifetime khat use		Lifetime cigarette use	
	Yes (%)	*P*-value	Yes (%)	*P*-value	Yes (%)	*P*-value
Age in years						
< 18	3 (0.6)	0.001	1 (0.2)	0.001	1 (0.2)	0.001
18–19.9	21 (4.4)		30 (6.2)		15 (3.1)	
20–21	43 (8.9)		61 (12.7)		38 (7.9)	
> 21	13 (2.7)		14 (2.9)		76 (15.8)	
Sex						
Male	1 (0.2)	0.001	80 (16.6)	0.001	49 (10.2)	0.001
Female	62 (12.9)		26 (5.4)		14 (2.9)	
Residency						
Urban	76 (15.8)	0.80	102 (21.2)	0.70	60 (12.5)	0.90
Rural	4 (0.8)		4 (0.8)		3 (0.6)	
Living years in the town						
1–5	33 (6.9)	0.006	43 (8.9)	0.001	24 (5.0)	0.004
6–10	10 (2)		15 (3.1)		7 (1.5)	
11–15	4 (0.8)		5 (1.0)		4 (0.8)	
16–20	17 (3.5)		19 (3.9)		14 (2.9)	
> 20	16 (3.3)		24 (5.0)		14 (2.9)	
Academic level						
level one	16 (3.3)	0.001	16 (3.3)	0.001	8 (1.7)	0.001
level two	13 (2.7)		29 (6.0)		17 (3.5)	
level three	28 (5.8)		38 (7.9)		20 (4.2)	
level four	23 (4.8)		23 (4.8)		18 (3.7)	
Currently living						
Alone	38 (7.9)	0.02	52 (10.8)	0.001	30 (6.2)	0.20
with family	22 (4.6)		26 (5.4)		19 (3.9)	
with peers	20 (4.2)		28 (5.8)		14 (2.9)	
Family monthly income (ETB)						
< 1000	8 (1.7)	0.001	8 (1.7)	0.001	5 (1.0)	0.001
1001–1500	15 (3.1)		14 (2.9)		8 (1.7)	
1501–2000	3 (0.6)		13 (2.7)		4 (0.8)	
> 2000	54 (11.2)		71 (14.7)		46 (9.5)	

### Lifetime and last month illegal substance use and its correlates

The prevalence of lifetime use of cannabis and cocaine was 2.5 and 7.2% respectively. Additionally, 1.2 and 3.3% of students used cannabis and cocaine in the last month respectively.

Variation across lifetime cannabis use observed in students’ living status and family monthly income. Lifetime cocaine use, the variation observed in sex, academic level, and living status (Table 3).

**Table 3:** Last month legal drugs use and its correlation with students and family characteristics among Ataye TVET college students, Ethiopia, 2019

Variables	Last month alcohol use		Last month khat use		Last month cigarette use	
	Yes (%)	*P*-value	Yes (%)	*P*-value	Yes (%)	*P*-value
Age in years (quartile)						
< 18	3 (0.6)	0.001	2 (0.4)	0.001	2 (0.4)	0.001
18–19.9	27 (5.6)		36 (7.5)		19 (3.9)	
20–21	45 (9.3)		67 (13.9)		43 (8.9)	
> 21	19 (3.9)		18 (3.7)		10()2.1	
Sex						
Male	74 (15.4)	0.001	92 (19.1)	0.001	56 (11.6)	0.001
Female	20 (4.1)		31 (6.4)		18 (3.7)	
Residency						
Urban	90 (18.7)	0.90	119 (24.7)	0.400	71 (9.1)	0.80
Rural	4 (0.8)		4 (0.8)		3 (0.6)	
Living years in the town						
1–5 years	41 (8.5)	0.01	50 (10.4)	0.001	29 (6.0)	0.01
6–10	12 (2.5)		15 (3.1)		7 (1.4)	
11–15	4 (0.8)		5 (1.0)		4 (0.8)	
16–20	18 (3.7)		24 (5.0)		19 (3.9)	
> 20	19 (3.9)		29 (6.0)		15 (3.1)	
Academic level						
level one	23 (4.8)	0.001	22 (4.6)	0.001	11 (2.3)	0.001
level two	17 (3.5)		33 (6.9)		21 (4.4)	
level three	28 (5.8)		44 (9.1)		23 (4.8)	
level four	26 (5.4)		24 (5.0)		19 (3.9)	
Currently living						
Alone	43 (8.9)	0.01	58 (12.0)	0.001	33 (6.9)	0.05
with family	27 (5.6)		33 (6.9)		22 (4.6)	
with peers	24 (5.0)		32 (6.6)		19 (3.9)	
Family income in ETB						
< 1000	8 (1.7)	0.001	8 (1.7)	0.001	5 (1.0)	0.001
1001_1500	17 (3.5)		21 (4.4)		13 (2.7)	
1501_2000	9 (1.9)		14 (2.9)		5 (1.0)	
> 2000	60 (12.5)		80 (16.6)		51 (10.6)	

Age difference had no variation for last month’s use of cannabis and cocaine (*p*-value = 0.11 vs. 0.6). All students with their different characteristics were equally practiced cannabis in the last month (*p* values were insignificant). Students’ residence, academic level, currently living status, and family monthly income were not statistically significant in association with last month’s cocaine use. This showed students with these predictor variables equally practiced cocaine use in the last month, (Table 4).

**Table 4:** Lifetime and last month illegal drugs use and its correlation with students and family characteristics among Ataye TVET college students, Ethiopia, 2019

Variables	Lifetime cannabis use		Lifetime cocaine use		Last month cannabis use		Last month cocaine use	
	Yes (%)	*P*-value	Yes (%)	*P*-value	Yes (%)	*p*-value	Yes (%)	*p*-value
Age in years (quartile)								
< 18	0 (0.00)	0.44	0 (0.0)	0.08	2 (0.4)	0.11	1 (0.2)	0.05
18–19.9	1 (0.2)		4 (0.8)		1 (0.2)		11 (2.3)	
20–21	3 (0.6)		10 (2.0)		7 (1.5)		19 (3.9)	
> 21	2 (0.4)		2 (0.4)		2 (0.4)		4 (0.8)	
Sex								
Male	4 (0.8)	0.40	15 (3.0)	0.001	8 (1.6)	0.20	31 (6.4)	0.001
Female	2 (0.4)		1 (0.2)		4 (0.8)		4 (0.8)	
Residence								
Urban	6 (1.2)	0.60	15 (3.0)	0.74	32 (6.64)	0.24	12 (2.4)	0.44
Rural	0 (0.00)		1 (0.2)		3 (0.6)		0 (0.0)	
Living years in the town								
1–5	2 (0.4)	0.60	3 (0.6)	0.03	2 (0.4)	0.20	16 (3.3)	0.20
6–10	2 (0.4)		3 (0.6)		2 (0.4)		6 (1.2)	
11–15	1 (0.2)		1 (0.2)		2 (0.4)		1 (0.2)	
16–20	0 (0.0)		4 (0.8)		3 (0.6)		5 (1.0)	
> 20	1 (0.2)		5 (1.0)		3 (0.6)		7 (1.5)	
Academic level								
level one	2 (0.4)	0.40	3 (0.6)	0.06	4 (0.8)	0.06	4 (0.8)	0.001
level two	1 (0.2)		6 (1.2)		3 (0.6)		12 (2.4)	
level three	1 (0.2)		7 (1.5)		1 (0.2)		13 (2.7)	
level four	2 (0.4)		0 (0.0)		4 (0.8)		6 (1.2)	
Currently living								
Alone	4 (0.8)	0.40	12 (2.4)	0.01	9 (1.8)	0.05	17 (3.5)	0.02
with family	2 (0.4)		1 (0.2)		3 (0.6)		7 (1.5)	
with peers	0 (0.0)		3 (0.6)		0 (0.0)		11 (2.3)	
Family income								
< 1000	1 (0.2)	0.50	1 (0.2)	0.20	1 (0.2)	0.05	2 (0.6)	0.08
1001_1500	1 (0.2)		3 (0.6)		2 (0.4)		9 (1.8)	
1501_2000	0 (0.0)		5 (1.0)		0 (0.0)		6 (1.2)	
> 2000	4 (0.4)		7 (1.5)		9 (1.8)		18 (3.6)	

### Associated factors of lifetime legal substance use

During bivariate analysis, a cut point of *p*-value less than 0.20 was used to export variables to multivariate analysis. These variables were exported to multivariable binary logistic regression. During multivariate analysis, six predictor variables became statistically significant factors of lifetime legal substances use (*P*-value < 0.05).

Among sociodemographic variables of the students, being male had a statistically significant association with lifetime legal substances use (AOR = 2.2 (95% CI: 1.23, 3.84). Males had two times higher odds to use legal substances in a lifetime (after joining college) as compared with females. Living more than 20 years in the town (Ataye) was almost four times higher odds of legal substances use at least once in a lifetime as compared with who lived 1–5 years, (AOR = 3.45, 95%CI: 1.18, 10.1). Students from divorced parents had four times higher odds to practice lifetime legal substance use as compared with married parents, (AOR = 4.1, 95%CI:1.78, 9.30). Having a substance user family was a predictor of lifetime legal substance use. The odds of experiencing lifetime legal substance use were 2.5 times higher among students, who had a substance user family than those who don’t have, (AOR = 2.5, 95%CI: 1.1, 5.8). Having an intimate friend who uses substance and easily availability of drugs in the Ataye town were also had a contribution to experience legal substances use in a lifetime, (AOR = 5.3, 95% CI: 2.6, 10.9), and (AOR = 2.3, 95% CI: 1.2, 4.4) respectively, (Table 5).

**Table 5:** Bivariate and multivariate backward logistic regression analysis to identify associated factors with lifetime drug use among Ataye TVET students, Ethiopia, 2019

Variables	Lifetime use		COR (95%CI)	COR ***p***-value	AOR (95% CI)	AOR ***P***-value
	Yes	No				
Sex						
Male	112	128	3.85 (2.55,5.81)	0.001	2.2 (1.23,3.84)	0.008
Female	45	198	1.00		1.00	
Years living in the town						
1–5	70	138	1.00		1.00	
6–10	19	77	0.49 (0.27, 0.87)	0.001	0.81 (0.33,2.00)	0.644
11–15	7	33	0.42 (0.18, 0.99)	0.031	0.5 (0.15,1.66)	0.260
16–20	29	63	0.91 (0.54,1.54)	0.12	0.53 (0.21,1.33)	0.180
> 20	32	15	4.21 (2.14, 8.28)	0.001	3.45 (1.18,10.1)	0.024
Parent marital status						
Married	88	288	1.00		1.00	
Widowed/widower	11	19	1.90 (0.87,4.13	0.029	1.13 (0.37,3.44)	0.830
Divorced	58	19	9.99 (5.65,17.67)	0.001	4.1 (1.78,9.3)	0.001
Having substance user family						
No	85	306	1.00		1.00	
Yes	72	20	12.96 (7.47,22.48)	0.001	2.5 (1.1,5.8)	0.032
Having intimate friend substance user						
No	69	300	1.00		1.00	
Yes	88	26	14.72 (8.84, 24.5)	0.001	5.3 (2.6,10.9)	0.001
Easily availability of substances						
No	43	261	1.00		1.00	
Yes	114	65	10.65 (6.83,16.59)	0.001	2.3 (1.2,4.4)	0.013

*Key*: *COR* crude odds ratio, *AOR* adjusted odds ratio

## Discussion

Almost a third of the study participants experienced lifetime substance use and one-fifth use substance in the last month. Among lifetime legal substance users, nearly one-fourth chewed khat. Almost 20 and 15 % use alcohol and cigarette after joining TVET College. Among last month’s legal substance users, almost 22 % chewed khat followed by alcohol drinking (16.6%), and cigarette smoking (15.3%).

Lifetime and last month smoking of cigarette were lower than studies from southern Iran [22] and Jimma University [23]. The possible explanation might be due to the health behavior of the students, and the target population’s age difference.

Only two studies; one from Hawassa University [18] and another study from Gondar University [19] reported a similar prevalence (around 24%) of lifetime khat chewing. The prevalence of lifetime khat chewing in the current study was lower than studies from Haramaya [24], and Jimma [23] universities. The possible reason might be due to the availability of khat in Haramaya and Jimma town is more prevalent than the current study area. Especially, Haramaya dwellers linked khat to the economy of the household and chewed khat as a habit [25]. This allows students to be easily exposed and practiced khat. The prevalence of lifetime khat chewing was higher than studies done among Addis Ababa University, and Debre Berhan University. A possible explanation for the high prevalence of lifetime khat chewing in Ataye extends to social and environmental differences. First, khat is cultivated around Ataye town that could make it easy to access by students. All these factors can contribute to the practice of khat chewing among Ataye TVET students.

Last month khat chewing prevalence was lower than the study from the same study area [17] and Hawassa University [14]. Except for the study from south Iran [22], both last month and lifetime drinking of alcohol was lower than the studies from Jimma, Gondar, Debre Berhan, Hawassa University, and Woreta town [23, 26–28]. This might be due to social desirability bias, and increased abstinence rate in the past 30 days. Also, since the data collections have been done inside the teaching classroom, those students with addiction behaviors may remain outside the classroom because of their academic and living lifestyle. This can potentially introduce sampling bias and result in a lower estimate of alcohol drinking [18].

Male students had two times higher odds to use legal substances in their lifetime (after joining college) as compared with female students. The association is in agreement with studies done in Haramaya University [20], and Jimma University [23]. This association might be male students use substances than females due to cultural and hormonal differences [29–31].

Having substance user friends and family had higher odds to be exposed to legal substances used in a lifetime as compared with who has no legal substances user friend and family. The association is supported by studies done in Addis Ababa University [27], Debre Berhan University [32], Hawassa University [18], Grate Accra metropolis [33], and Woreta Town [28]. This might be due to, they may let students familiarize substances and adopt utilization thereby reducing the subjective norm and perceived risk perception of students [34]**.** Being from a divorced family also had a significant contribution to lifetime legal substance use as compared with students from married families. The study from Kuwait supports this association [35]. Many factors increase a young person’s likelihood of substance use. Among them, parental divorce is the main factor. Parental conflicts and lack of supervision from parents is a known factor for young’s health problem [36–39].

Easily availability of the substances was a statistically significant factor for lifetime substance use as compared with students from less substance accessibility areas. The possible reason might be, increased substance availability is associated with increased use [34]. In the study area, licit drugs are easily available; but not known about illicit drugs (cannabis and cocaine). At the study area (Ataye) and catchment areas, the community cultivates khat and this may put the study subjects at a greater risk of exposure.

Living 20 years and above in Ataye town had higher odds to be exposed to the lifetime legal substances as compared with those who lived 1–5 years. This might be due to; living more years in one town might increase the exposure of the study participants to substances. More years needed to assimilate the community habit of substance use; even if it is two decades.

### Generalizability

The external validity of the study was managed during a sample size calculation, sampling procedures, and techniques, training of data collectors, and data quality control sections.

### Limitations of the study

There are several limitations to the present study. Firstly, self-reported substance use is likely underreported perhaps because of social desirability bias. Secondly, there is no means to verify the self-reports by biomarkers. Thirdly, the association shown from the cross-sectional study lacks temporal association. Lastly, the questionnaire used to assess the outcome and independent variables were not validated.

## Conclusions

The prevalence of last month and lifetime legal substance use at Ataye Technical and Vocational Education and Training **(**TVET**)** college students were analogous with most studies done in Ethiopia. It is advisable if the college administrators work together with town administrators to mitigate the problem including closing substance use houses around the school. Overall, Substance use among adolescents should get further emphasis to lower the prevalence.

## Data Availability

The datasets used and/or analyzed during the current study are available from the corresponding author on reasonable request.

## Abbreviation

TVET: Technical and vocational education and training
